# Muscularity and combatives training experience in social judgments among police cadets

**DOI:** 10.3389/fpsyg.2026.1831520

**Published:** 2026-10-21

**Authors:** Zuwei Mi, Yike Li, Zhengyang Mei, Lunxin Chen, Hengzhi Deng, Mingyue Yin, Xing Zhang, Yufei Tao, Hansen Li

**Affiliations:** 1 College of Physical Education, Guizhou University of Engineering Science, Guizhou, China; 2 College of Physical Education, Southwest University, Chongqing, China; 3 School of Physical Education and Sports, Central China Normal University, Wuhan, China; 4 Faculty of Sports and Exercise Science, University of Malaya, Kuala Lumpur, Malaysia; 5 School of Athletic Performance, Shanghai University of Sport, Shanghai, China; 6 Department of Physical Education and Sport, Faculty of Sport Sciences, University of Granada, Granada, Spain; 7 School of Sport Training, Chengdu Sport University, Chengdu, Sichuan, China; 8 School of Physical Education, Sichuan Agricultural University, Ya’an, China

**Keywords:** body image, exercise, martial arts, sports, stereotype

## Abstract

**Introduction:**

Muscular appearance may influence judgments of others’ physical abilities and social qualities. However, whether these judgments vary according to observers’ combatives training experience remains unclear. This study examined associations between target muscularity, police cadets’ self-reported combatives experience, and judgments of competence, warmth, and morality.

**Methods:**

Participants were 308 cadets from a Chinese police university. Each participant rated one male target followed by one female target. Within each target set, muscularity was independently randomized across four intended image levels. Participants evaluated 14 attributes on 11-point scales. Separate covariate-adjusted models were fitted for the male and female targets and included muscularity, self-reported combatives experience, and their interaction. Sensitivity analyses removed the interaction term. Holm correction was applied across outcomes.

**Results:**

In both model specifications, greater muscularity was associated with higher perceived strength and resistance to blows for the male target and with higher perceived strength, explosiveness, and resistance to blows for the female target. Models without the interaction also identified associations with male-target explosiveness and enthusiasm and female-target fighting skill. No morality outcome remained significant after correction in either model. The full models detected no statistically significant training-group differences or muscularity-by-experience interactions. However, the imbalance between experienced and inexperienced participants (280 versus 28) limited the precision of these estimates. Although four female-image conditions were assigned, the manipulation check distinguished only two broad perceived-muscularity groups.

**Discussion:**

Muscularity was most consistently associated with impressions of physical capability, while several findings depended on model specification. Evidence that combatives experience was related to these judgments was inconclusive. Interpretation is limited by the self-reported training measure, the highly unequal experience groups, the fixed male–female target order, and the limited perceptual separation among the female images. These findings do not establish that combatives training changes social judgments.

## 1 Introduction

Muscle supports metabolism (Wolfe, 2006), health (Burini and Maestá, 2012; Mijnarends et al., 2018), strength (Kanehisa et al., 1994; Reed et al., 1991), and physical performance (Hayashida et al., 2014; Suchomel et al., 2016). Its visible appearance also carries social meaning. Muscularity has been linked to male attractiveness (Sell et al., 2017), feelings of confidence and power (Grogan and Richards, 2002), and men’s perceptions of their own sexual attractiveness (Frederick and Haselton, 2007). These links raise a broader question: which qualities do observers infer from another person’s muscular appearance?

The desire for muscularity is well documented (Morrison et al., 2003) and can involve unhealthy extremes (Olave et al., 2021). Body ideals also vary by gender: men often seek larger, stronger bodies, while women commonly seek slimmer bodies (Davis et al., 2005; McCreary and Sasse, 2000), with increasing emphasis on a fit, toned appearance (Campos et al., 2021; Rodgers et al., 2018). These ideals are visible on social media: a content analysis found that male fitspiration subjects were often muscular or very muscular, while female subjects were typically thin and toned (Carrotte et al., 2017).

Muscularity can signal strength and competence, but it can also attract stereotypes of violence or low intelligence (He et al., 2025). Muscular action-film characters illustrate its cultural link with fighting ability (Morrison and Halton, 2009), although attitudes toward muscularity vary across cultural settings (Kanayama et al., 2012). Men’s muscularity and strength have also been linked to their own perceived fighting ability (Muñoz-Reyes et al., 2019). Less clear is how muscular appearance relates to observers’ judgments across physical and social qualities.

We organized these judgments using the morality–warmth–competence framework (Brambilla and Leach, 2014; Brambilla et al., 2011), which extends the warmth–competence model (Cuddy et al., 2009; Fiske et al., 2007). This distinction allows us to ask whether muscular appearance mainly informs judgments of physical capability or also relates to judgments of character and interpersonal qualities.

Combatives experience may be relevant because fighting requires technique, timing, coordination, and physical capacity. A still image reveals little about several of these qualities. Observers with live-sparring experience may therefore use muscular appearance differently when judging strength, fighting skill, or resistance to blows. We examined both overall rating differences between experience groups and whether their ratings followed different patterns across muscularity levels. Morality and warmth ratings allowed us to examine the breadth of these impressions. Dangerousness and threat were not measured.

Police cadets provide a relevant setting for this question. Their future work may involve physical confrontation, and fair person perception is an important concern in policing (Dukes and Gaither, 2017; Najdowski et al., 2015). Police education also provides access to students with live-sparring experience. We therefore examined muscularity-related judgments and self-reported training experience in this population.

We assessed 14 attributes covering physical competence, warmth, and morality. Each attribute was analyzed separately. Our hypotheses were:

(H1) Muscularity is associated with various stereotype judgments, such as combative ability.

(H2) The association between muscularity and stereotype judgments differs between participants with and without self-reported combatives training experience.

## 2 Materials and methods

### 2.1 Participants

We recruited students from a police university in Chongqing, China. Teachers only distributed the questionnaire and did not check completion status or inspect responses. Participation was voluntary and fully anonymous, with no rewards or penalties. The questionnaire explained that students could withdraw at any time, that responses were for research only, and that individual-level data would not be displayed. Students could accept or decline participation before proceeding.

The Ethics Committee of Chengdu Sport University approved the study (Code: 2026/NO. 78). The authorized survey population consisted of general university students, including students from both our university and other institutions, and recruitment was conducted through both online and offline survey channels.

Because that university did not offer the target major, data collection took place at the police college in Chongqing. In accordance with the special administrative requirements of the police college, prior to the commencement of the survey, the study was formally reported to the institution’s department responsible for science and technology administration, with the research design and relevant explanatory materials submitted for review. The survey was conducted only after permission had been granted by the institution. This procedure was adopted because the college did not have an institutional ethics committee at the time; therefore, the administrative reporting and approval process was regarded as an alternative institutional oversight measure.

Students were eligible if they were in good health, with no condition restricting physical activity or combatives training. Incomplete questionnaires and clearly abnormal response patterns, such as invariant responding, were excluded. The final sample comprised 308 students. Participants were required to provide informed consent before completing the questionnaire.

An a priori G*Power calculation used *f* = 0.25, the conventional medium-effect benchmark, with *α* = 0.05, power = 0.80, and numerator df = 3 for the four-level muscularity test. It yielded *N* = 179 under balanced allocation. The effect size was a planning assumption, with no estimate from pilot data or a closely matched study. The calculation did not account for testing 14 outcomes, smaller effects, or the realized imbalance in training experience. Exceeding this sample-size target therefore does not establish adequate power for every analysis.

### 2.2 Design and procedure

We used an image-based design (Cao and Huang, 2023; Li et al., 2022; Shici et al., 2025). Each participant rated one male and one female target. Wenjuanxing independently randomized the muscularity level within each target set. Target order was fixed: all students rated the male target first and the female target second, with no counterbalancing. The two target sets were analyzed separately; the design did not test target-sex differences or target sex as a moderator.

The images were generated around October 2025 using the web version of ChatGPT with GPT-5. For each target sex, the prompt requested: “Generate a realistic photograph showing a male/female combat athlete at four distinct levels of muscularity, ranging from lean to highly muscular. Keep the athlete’s pose, face, clothing, camera angle, and solid-color background unchanged; vary only muscularity.” The authors selected the clearest and most realistic sets from more than 20 candidate images. Each set depicted one identity at four intended muscularity levels, with facial appearance, skin tone, hairstyle, pose, clothing, lighting, and background kept as constant as possible. Faces remained visible. Chinese-appearing identities were chosen for relevance to the sample. Evidence that stimulus ethnicity matters for body-size judgments (Ridley et al., 2025) offers an indirect rationale; that study did not examine morality, warmth, or competence ratings.

No independent pretest or expert review was conducted because limited access and resources allowed only one survey wave. Validation used the perceived-muscularity ratings collected in the main sample. The female set distinguished two broad groups, levels 1–2 and levels 3–4; neither pair differed internally. We retained the four randomized image conditions in the analyses, while limiting interpretation of a four-level perceptual progression. Detailed manipulation-check results appear in Section 3.2 (Figure 1).

**Figure 1:**
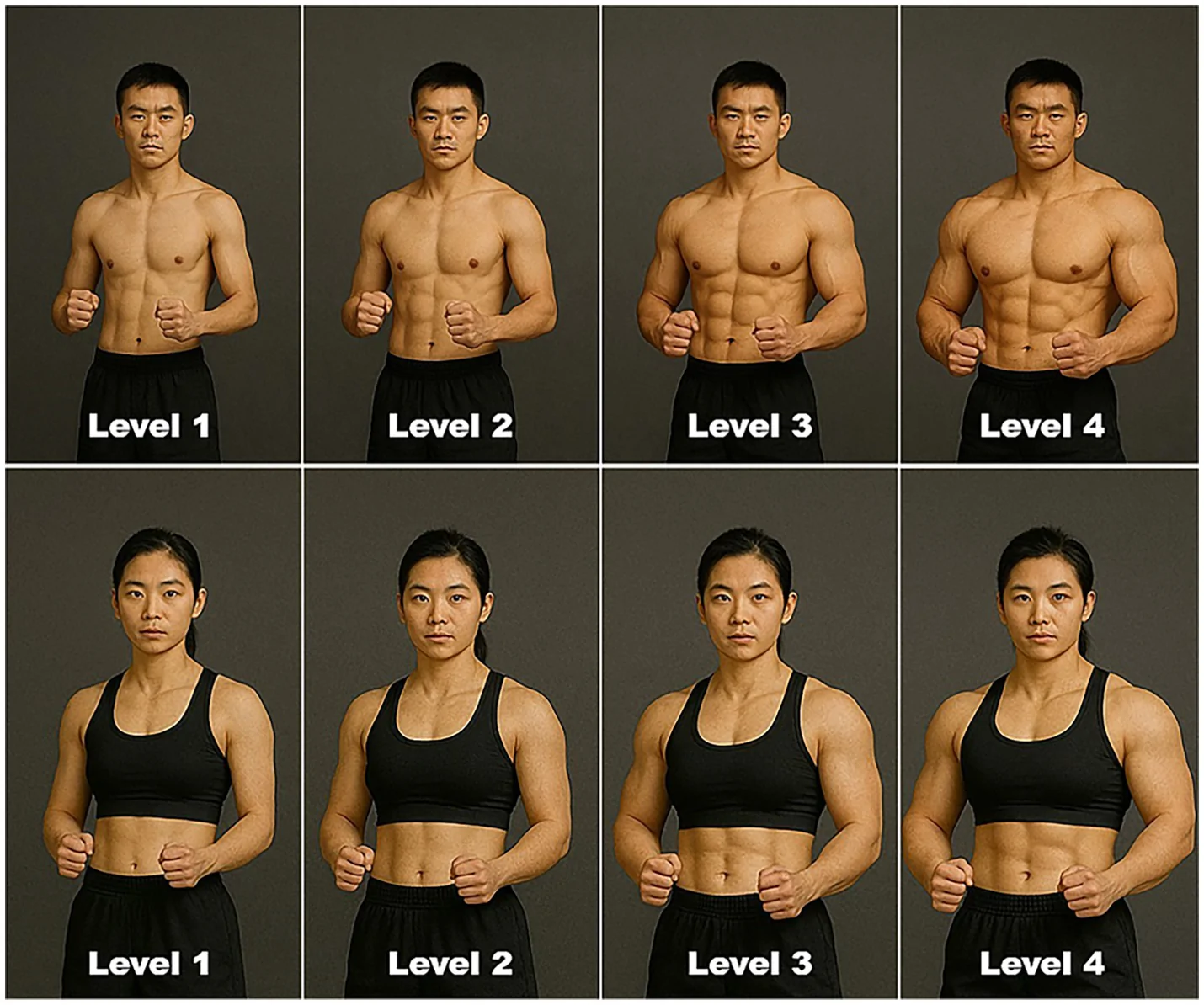
Male and female AI-generated targets at four intended muscularity levels. Each target set depicts the same identity. The female manipulation check distinguished two broad perceived-muscularity groups.

Students completed the questionnaire on tablets during classroom sessions. Image exposure time was not controlled. We therefore describe the ratings as image-based impressions, without assuming that they capture rapid or automatic first impressions.

### 2.3 Measurements

#### 2.3.1 Focal variables and rating items

Combatives training experience was assessed by asking whether participants had completed live-sparring training or the university’s “Police Defense and Control” course (yes/no). This self-reported variable describes training history and cannot establish a causal training effect.

Participants completed semantic-differential ratings (Huang et al., 2012) covering the three judgment categories, consistent with prior physique-stereotype research (Baker and Florack, 2021). Each item used an 11-point numeric scale (Hjermstad et al., 2011) between opposing adjectives. Higher scores indicated more positive evaluations. The items were:

The 11-point scale provided a neutral midpoint. The items were analyzed separately because they had not been validated as combined dimension scores.

(1) Morality dimension.

- Law-abiding: non-law-abiding → law-abiding.
- Rule-compliance: disregards rules → complies with rules.
- Honesty: deceitful → honest.
- Justice: treats others unfairly → treats others fairly.

(2) Warmth dimension.

- Friendliness: hostile → friendly.
- Enthusiasm: cold/indifferent → warm/enthusiastic.
- Approachability: hard to approach → easy to approach.
- Cooperativeness: hard to cooperate with → easy to cooperate with.

(3) Competence dimension.

- Strength: weak → strong.
- Explosiveness: lacks explosiveness → highly explosive.
- Fighting skill: poor at fighting → good at fighting.
- Endurance: insufficient endurance → ample endurance.
- Coordination: uncoordinated → coordinated.
- Resistance to blows: does not tolerate strikes → tolerates strikes.

#### 2.3.2 Manipulation check

To verify the efficacy of the muscularity manipulation, participants also rated each target’s perceived muscularity on the same 11-point NRS.

#### 2.3.3 Participant characteristics and covariates

We recorded age, participant sex, year in school, monthly household income, major, BMI, and self-rated strength.

Covariates were selected before examining outcome associations. Demographic and educational characteristics represented differences that could relate to training history and judgments. BMI and self-rated strength represented participants’ own physical reference points. Prior research has examined observer BMI (Baker and Florack, 2021) and professional background (Cassiano et al., 2022) in physique-related judgments. Adjustment addressed participant heterogeneity; covariate coefficients were not interpreted causally.

Self-rated strength was measured with “How would you rate your own strength level?” Responses used 11 ordered points from 1 (“very poor”) to 11 (“very good”). This single subjective item was included as a covariate, and its associations were treated as exploratory.

### 2.4 Statistical analysis

#### 2.4.1 Model specification

We fitted Gaussian identity-link generalized linear models to the 11-point ratings. Except for male-target fighting skill (absolute skewness = 1.08), outcomes had absolute skewness below 1 and absolute kurtosis below 3. This specification was chosen for the approximately continuous ratings (Kim, 2013) and expresses adjusted differences in the original scale units.

The full models included muscularity as a four-level categorical factor, combatives experience as a two-level factor, their interaction, and all seven covariates. These models addressed the original hypotheses. Separate models were fitted for each outcome and target set, with one observation per participant in each model.

#### 2.4.2 Predictor coding and model checks

Muscularity used sum-to-zero contrasts: levels 1–4 were coded (1, 0, 0), (0, 1, 0), (0, 0, 1), and (−1, −1, −1). Experience was coded −1 for no and +1 for yes. The three interaction terms were the products of these contrasts. In the full models, the Type III muscularity test compares adjusted means while giving the two experience groups equal weight. Thus, the 28 inexperienced participants receive half the weight in this contrast. The experience test compares groups averaged equally over the four image levels, and the interaction test assesses whether the muscularity pattern differs between groups.

Age, BMI, and self-rated strength entered as numeric terms. Year in school used numeric codes 1–3; income used ordered codes 1–6 for ≤5,000, 5,001–10,000, 10,001–15,000, 15,001–20,000, 20,001–25,000, and >25,000 RMB per month. The year and income terms assume a constant rating difference per category step. Participant sex used one dummy variable (male = 0, female = 1). Major used eight dummies for nine categories, with Traffic Management Engineering as the reference, after combining two equivalent forensic-science labels. The full models contained 22 parameters: one intercept, seven focal-predictor terms, five numeric covariates, one sex dummy, and eight major dummies. Residual degrees of freedom were 308–22 = 286.

The design matrices were full rank. Most column-wise variance inflation factors were modest, but some muscularity and interaction contrast columns exceeded 5 (maximum = 6.47). These values indicate overlap among predictors without rank deficiency (Shrestha, 2020).

#### 2.4.3 Statistical tests and sensitivity analyses

We used two-tailed tests with *α* = 0.05. Holm correction controlled multiple testing (Aickin and Gensler, 1996) across the 14 outcomes within each target set, separately for muscularity, experience, and interaction tests. Exploratory self-rated-strength tests formed a separate 14-outcome family. Pairwise muscularity comparisons used Holm correction within each outcome. Comparisons following nominal omnibus findings were reported as exploratory when the omnibus test did not survive across-outcome correction.

We report raw and adjusted *p* values, adjusted differences with 95% Wald confidence intervals, and the Wald-based index partial *η*^2^*p* = *χ*^2^/(*χ*^2^ + dfresidual). The full models used dfresidual = 286. Gaussian-model Wald tests used maximum-likelihood dispersion, calculated as residual sum of squares divided by *N*. Tables and figures identify which model and correction each result uses.

We then refitted all 14 outcomes in each target set without the muscularity × experience interaction, retaining both main predictors and every covariate. These models had 19 parameters and 289 residual degrees of freedom. We used the same method to estimate dispersion and applied Holm correction across the 14 muscularity tests within each target set. This sensitivity analysis assesses whether findings persist under a common muscularity pattern across experience groups. Removing the interaction changes the model assumption as well as the estimation of muscularity differences; it is not a direct reweighting of the full-model results. A non-significant interaction does not confirm that the muscularity–rating relationship is the same in both groups.

#### 2.4.4 Common method check

Harman’s single-factor test (Fuller et al., 2016) was used as a descriptive check of shared variance among questionnaire responses. This check alone cannot rule out common method bias.

## 3 Results

### 3.1 Participant characteristics

The sample comprised 308 cadets, including 271 men (88.0%), with a mean age of 19.35 years (SD = 0.77) and BMI of 22.00 (SD = 2.35). Most were sophomores (82.8%), and nine major categories were represented. Self-rated strength averaged 6.02 (SD = 1.67; range = 1–11). Training experience was highly unbalanced: 280 participants reported experience and 28 did not. Image-level counts ranged from 63 to 85. Within image levels, inexperienced versus experienced counts ranged from 5–8 versus 62–76 for the male target and 2–12 versus 61–76 for the female target. Table 1 provides participant characteristics and allocations.

**Table 1:** Participant characteristics and allocation of image materials.

Variable	Frequency	Percent	Mean (SD)
Sex			
Male	271	88.0	
Female	37	12.0	
Age (years)			19.35 (0.77)
BMI (kg/m^2^)			22.00 (2.35)
Year in school			
Freshman	18	5.8	
Sophomore	255	82.8	
Junior	35	11.4	
Household income (RMB/month)			
0–5,000	42	13.6	
5,001–10,000	106	34.4	
10,001–15,000	86	27.9	
15,001–20,000	46	14.9	
20,001–25,000	20	6.5	
>25,000	8	2.6	
Major			
Political Work in Public Security	17	5.5	
Traffic Management Engineering	30	9.7	
Police Command & Tactics	24	7.8	
Foreign-related Policing	18	5.8	
Cybersecurity & Law Enforcement	46	14.9	
Forensic Science & Technology	65	21.1	
Tactics Studies	6	1.9	
Criminal Investigation	35	11.4	
Public Security	67	21.8	
Combatives experience			
No	28	9.1	
Yes	280	90.9	
Male target (muscularity level)			
Level 1	81	26.3	
Level 2	75	24.4	
Level 3	70	22.7	
Level 4	82	26.6	
Female target (muscularity level)			
Level 1	85	27.6	
Level 2	85	27.6	
Level 3	75	24.4	
Level 4	63	20.5	
Self-rated strength (1–11)			6.02 (1.67)

### 3.2 Manipulation and response checks

Perceived muscularity increased across the four male images (M [SD]: 7.56 [1.35], 8.52 [1.12], 9.51 [1.09], and 10.07 [0.97]), with every adjacent pair differing in the adjusted comparisons (Figure 2a). Female-image means also increased (7.52 [1.67], 8.32 [1.73], 9.28 [1.30], and 9.60 [1.30]), but the adjusted comparisons distinguished two broad groups: levels 1–2 and levels 3–4 (Figure 3a). Levels 1 and 2 did not differ, nor did levels 3 and 4. The female set therefore supports a two-group perceptual distinction across four assigned image conditions.

**Figure 2:**
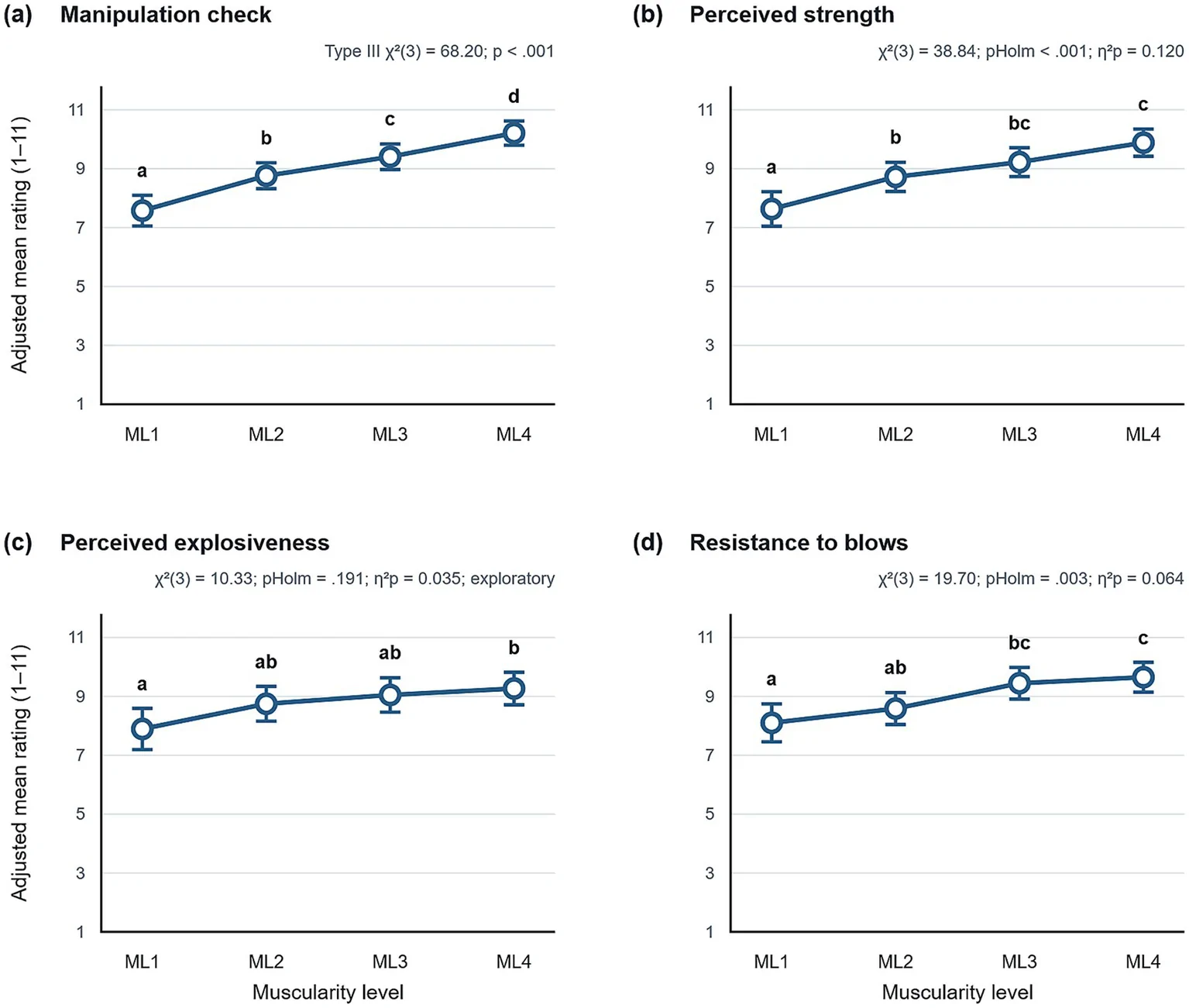
Male-target ratings from the full models. Panels show perceived muscularity, strength, explosiveness, and resistance to blows. ML1–ML4 are the assigned image levels. Points are adjusted means and bars are 95% confidence intervals, with experience groups weighted equally. Shared letters indicate no significant pairwise difference after within-panel Holm correction. Male-target explosiveness did not survive the separate correction across 14 outcomes and is shown as exploratory.

**Figure 3:**
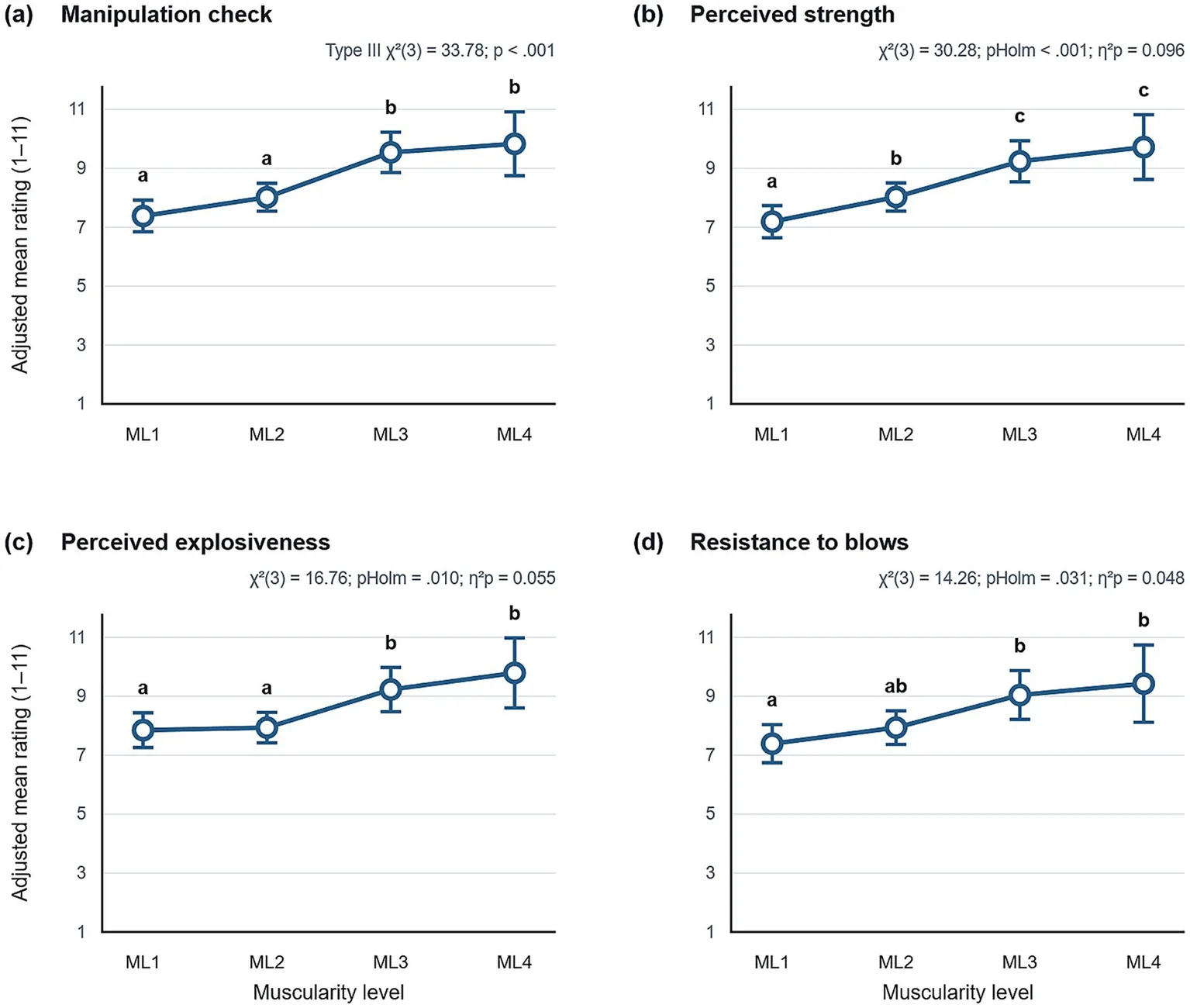
Female-target ratings from the full models. Panels show perceived muscularity, strength, explosiveness, and resistance to blows. ML1–ML4 are the assigned image levels. Points are adjusted means and bars are 95% confidence intervals, with experience groups weighted equally. Shared letters indicate no significant pairwise difference after within-panel Holm correction. The three displayed competence outcomes survived correction across 14 outcomes. The manipulation check distinguished levels 1–2 from levels 3–4.

The first unrotated factor accounted for 30.42% of questionnaire variance in Harman’s test. No single factor accounted for most of the variance, although this result does not exclude shared response influences.

### 3.3 Results from models with the interaction

This section reports the full models, including the muscularity × experience interaction. Their muscularity tests average the two experience groups equally. Section 3.4 reports the models without the interaction.

For the male target, muscularity was nominally associated with strength, explosiveness, and resistance to blows (Table 2). After correction across 14 outcomes, strength [Wald *χ*^2^(3) = 38.839, pHolm < 0.001, partial *η*^2^*p* = 0.120] and resistance to blows [*χ*^2^(3) = 19.697, pHolm = 0.003, partial *η*^2^*p* = 0.064] remained significant. Explosiveness did not survive correction (raw *p* = 0.016, pHolm = 0.191). No morality or warmth item showed a nominal muscularity association.

**Table 2:** Full-model results for male-target judgments.

Outcome	Statistic	Combatives	Muscularity	Combatives × Muscularity
Law-abiding	Wald *χ*^2^	0.340 (*p* = 0.560)	1.851 (*p* = 0.604)	0.842 (*p* = 0.839)
Rule-compliance		0.824 (*p* = 0.364)	0.636 (*p* = 0.888)	0.466 (*p* = 0.926)
Honesty		1.062 (*p* = 0.303)	4.171 (*p* = 0.244)	3.569 (*p* = 0.312)
Justice		1.187 (*p* = 0.276)	4.406 (*p* = 0.221)	3.705 (*p* = 0.295)
Friendliness		1.367 (*p* = 0.242)	2.903 (*p* = 0.407)	1.849 (*p* = 0.604)
Enthusiasm		0.181 (*p* = 0.671)	4.677 (*p* = 0.197)	4.776 (*p* = 0.189)
Approachability		1.053 (*p* = 0.305)	2.883 (*p* = 0.410)	3.258 (*p* = 0.354)
Cooperativeness		1.218 (*p* = 0.270)	1.291 (*p* = 0.731)	1.159 (*p* = 0.763)
Strength		0.101 (*p* = 0.750)	**38.839 (*p* < 0.001)**	1.296 (*p* = 0.730)
Explosiveness		0.444 (*p* = 0.505)	**10.330 (*p* = 0.016)**	2.862 (*p* = 0.413)
Fighting skill		0.458 (*p* = 0.499)	7.069 (*p* = 0.070)	3.051 (*p* = 0.384)
Endurance		0.476 (*p* = 0.490)	3.956 (*p* = 0.266)	1.653 (*p* = 0.647)
Coordination		1.982 (*p* = 0.159)	5.365 (*p* = 0.147)	1.080 (*p* = 0.782)
Resistance to blows		0.012 (*p* = 0.914)	**19.697 (*p* < 0.001)**	1.255 (*p* = 0.740)

Full models included the muscularity × experience interaction and all seven covariates. Wald tests have df = 1 for experience and df = 3 for muscularity and the interaction. The muscularity test gives the experience groups equal weight. Raw *p* values are shown; Table 3 reports correction across 14 outcomes, and Supplementary Table S3 reports models without the interaction. Bold data highlights results with uncorrected *p*-values less than 0.05.

Pairwise differences in perceived strength and resistance to blows were concentrated between lower and higher image levels. Figure 2 shows within-outcome comparisons, including exploratory explosiveness contrasts; Table 3 reports the across-outcome correction.

**Table 3:** Muscularity tests from the full models with correction across outcomes.

Target	Outcome	Wald *χ*^2^(3)	Raw *p*	pHolm	Partial *η*^2^*p*	Inference
Male	Strength	38.839	<0.001	<0.001	0.120	Retained
Male	Explosiveness	10.330	0.016	0.191	0.035	Not retained
Male	Resistance to blows	19.697	<0.001	0.003	0.064	Retained
Female	Strength	30.277	<0.001	<0.001	0.096	Retained
Female	Explosiveness	16.761	0.001	0.010	0.055	Retained
Female	Fighting skill	9.973	0.019	0.207	0.034	Not retained
Female	Resistance to blows	14.265	0.003	0.031	0.048	Retained

All statistics are from models containing the interaction. pHolm adjusts 14 muscularity tests separately within each target set. Partial *η*^2^*p* = *χ*^2^/(*χ*^2^ + 286). “Retained” means significant after this correction; it does not compare target sexes. Results without the interaction appear in Supplementary Table S3.

For the female target, muscularity was nominally associated with strength, explosiveness, fighting skill, and resistance to blows (Table 4). Strength [Wald *χ*^2^(3) = 30.277, pHolm < 0.001, partial *η*^2^*p* = 0.096], explosiveness [*χ*^2^(3) = 16.761, pHolm = 0.010, partial *η*^2^*p* = 0.055], and resistance to blows [*χ*^2^(3) = 14.265, pHolm = 0.031, partial *η*^2^*p* = 0.048] survived correction. Fighting skill did not (raw *p* = 0.019, pHolm = 0.207). No morality or warmth item showed a nominal muscularity association. Pairwise differences for the retained outcomes were concentrated between more widely separated image levels (Figure 3).

**Table 4:** Full-model results for female-target judgments.

Outcome	Statistic	Combatives	Muscularity	Combatives × Muscularity
Law-abiding	Wald *χ*^2^	0.068 (*p* = 0.794)	2.841 (*p* = 0.417)	1.761 (*p* = 0.623)
Rule-compliance		0.215 (*p* = 0.643)	2.782 (*p* = 0.426)	1.715 (*p* = 0.634)
Honesty		0.135 (*p* = 0.713)	2.402 (*p* = 0.493)	0.966 (*p* = 0.809)
Justice		0.257 (*p* = 0.612)	2.394 (*p* = 0.495)	0.562 (*p* = 0.905)
Friendliness		0.000 (*p* = 0.998)	2.959 (*p* = 0.398)	0.868 (*p* = 0.833)
Enthusiasm		0.077 (*p* = 0.781)	2.507 (*p* = 0.474)	1.438 (*p* = 0.697)
Approachability		0.015 (*p* = 0.902)	0.800 (*p* = 0.849)	0.851 (*p* = 0.837)
Cooperativeness		0.017 (*p* = 0.897)	5.661 (*p* = 0.129)	4.097 (*p* = 0.251)
Strength		0.554 (*p* = 0.457)	**30.277 (*p* < 0.001)**	2.790 (*p* = 0.425)
Explosiveness		1.439 (*p* = 0.230)	**16.761 (*p* = 0.001)**	4.234 (*p* = 0.237)
Fighting skill		1.780 (*p* = 0.182)	**9.973 (*p* = 0.019)**	4.586 (*p* = 0.205)
Endurance		0.023 (*p* = 0.881)	4.366 (*p* = 0.225)	1.442 (*p* = 0.696)
Coordination		0.242 (*p* = 0.623)	3.058 (*p* = 0.383)	1.491 (*p* = 0.684)
Resistance to blows		0.300 (*p* = 0.584)	**14.265 (*p* = 0.003)**	2.584 (*p* = 0.460)

Full models included the muscularity × experience interaction and all seven covariates. Wald tests have df = 1 for experience and df = 3 for muscularity and the interaction. The muscularity test gives the experience groups equal weight. Raw p values are shown; Table 3 reports correction across 14 outcomes, and Supplementary Table S3 reports models without the interaction. Bold data highlights results with uncorrected *p*-values less than 0.05.

Neither overall training-group differences nor muscularity × experience interactions were statistically detectable in either target set. Adjusted experienced-minus-inexperienced differences ranged from −0.64 to 0.58 scale points, and all 95% confidence intervals included zero (Supplementary Table S1). Partial *η*^2^*p* ranged from 0.000 to 0.007 for experience and 0.002 to 0.016 for interactions. These estimates do not establish equivalence between the highly unbalanced groups.

### 3.4 Results from models without the interaction

All five muscularity associations retained in the full models also survived correction in the models without the interaction (Supplementary Table S3). For the male target, strength and resistance to blows remained significant (both pHolm < 0.001), and explosiveness (pHolm < 0.001) and enthusiasm (pHolm = 0.014) also survived correction. For the female target, strength, explosiveness, and resistance to blows remained significant (all pHolm < 0.001), and fighting skill also survived correction (pHolm = 0.035). Male-target fighting skill remained non-significant (raw *p* = 0.119, pHolm = 0.596). No morality item survived correction in either target set.

The most consistent findings therefore concerned physical capability. Male-target explosiveness and enthusiasm and female-target fighting skill depended on model specification. In particular, the enthusiasm result limits a general claim that muscularity was unrelated to warmth ratings.

### 3.5 Exploratory covariate findings

Self-rated strength was positively associated with seven male-target morality or warmth ratings and two female-target ratings at the nominal level (Supplementary Table S2). After correction across 14 outcomes, only male-target law-abidingness (pHolm = 0.049) and friendliness (pHolm = 0.037) remained significant. These exploratory covariate findings do not establish causal effects.

## 4 Discussion

### 4.1 Summary of findings

Muscularity was most consistently associated with perceived physical capability: both model specifications retained strength and resistance to blows for the male target and strength, explosiveness, and resistance to blows for the female target. Three additional associations appeared only after removing the interaction, including male-target enthusiasm. No morality item survived correction in either model. These findings provide partial support for H1. The full models did not detect the interaction proposed in H2, but the small inexperienced group limits what can be concluded about training-related differences.

### 4.2 Physical capability and social judgments

The physical-capability pattern is consistent with evidence linking muscle cross-sectional area to force (Jones et al., 2008), lower-limb muscularity to jump performance (Stephenson et al., 2015), and body mass and strength to punch impact (Dunn et al., 2022). Neck strength has also been examined in relation to head acceleration and concussion risk (Silverman et al., 2024). These links offer a possible basis for observers’ judgments. However, the ratings do not establish the actual abilities of the pictured targets.

Fighting skill was less consistent than strength or resistance to blows. For the male target, it was non-significant in both the full model (raw *p* = 0.070, pHolm = 0.767) and the model without the interaction (raw *p* = 0.119, pHolm = 0.596). For the female target, it survived correction only without the interaction. Still images may convey apparent strength more clearly than technique or timing, although we did not measure which cues participants used.

Body shape has been linked to sexual impressions and attractiveness (Oswald et al., 2022), and body weight to negative stereotypes (Harriger et al., 2010). Related studies have also reported associations between men’s body shape and stereotypical perceptions of their warmth and competence (Baker and Florack, 2021). Our morality results may differ because a policing-related task invites different judgments from a romantic context. Cultural beliefs or professional-ethics education could also contribute, but neither explanation was tested. Facial information offers another possibility: Dixson et al. (2024) examined facial cues to dominance and trustworthiness, and broader research documents face-based judgments (Zebrowitz and Montepare, 2008). Nearly constant faces may have provided stable cues across our images. These remain possible explanations, and the single-item measures may also have limited sensitivity. Warmth warrants separate caution because male-target enthusiasm was significant in one model specification.

### 4.3 Training experience and exploratory findings

The absence of detectable training-group differences may partly reflect the limited information in posed images. Expertise advantages in combat-sport anticipation are strongest when stimuli preserve dynamic or in-situ information (Zhang et al., 2022), while a study using static images found no top-down expertise advantage in action perception (Harrison et al., 2024). This offers one possible explanation, but evidence from other tasks does not establish the mechanism here. The binary experience measure and only 28 inexperienced participants also limit interpretation; the findings cannot show that training changes or leaves judgments unchanged.

Self-rated strength was associated with two male-target ratings after correction. These findings may reflect perceived capability, response style, or unmeasured participant characteristics. Threat-management theory (Neuberg et al., 2011) offers a possible direction for future research, but safety and trust were not measured here. The present findings support further study of observer characteristics and do not establish a psychological pathway.

### 4.4 Contributions and future applications

The study identifies a recurring link between muscular appearance and perceived physical capability in police cadets, while showing that some individual judgments depend on model specification. The same-identity image sets provide a useful starting point for further studies with more identities and independently validated stimuli. For policing education, a next step would be to test whether similar appearance-based judgments occur in realistic scenarios and influence decisions. The present data do not establish the effectiveness of any educational intervention.

### 4.5 Limitations

The sample came from one Chinese police academy, was predominantly male, and covered a narrow age and educational range. Training experience was self-reported and highly unbalanced (280 versus 28), leaving small inexperienced cells and uncertain group comparisons. Unmeasured differences between training groups also prevent causal conclusions about experience. Some muscularity findings changed when the interaction was removed, and the sample-size calculation did not account for the 14 outcomes. Replication with more balanced experience groups is needed.

The design used one static identity per target sex. All participants rated the male target first, so target sex was inseparable from presentation position. Contrast or carryover could influence the second ratings, and separate analyses do not remove this limitation. Future studies should use more identities and randomize or counterbalance target order. Because exposure time was uncontrolled, the ratings cannot be assumed to represent rapid or automatic impressions.

Muscularity was not varied independently of body-fat distribution, and validation relied on the main sample without an independent pretest or expert review. The female images distinguished two broad perceived-muscularity groups, limiting claims about a four-level progression. The judgment items and self-rated-strength item were not validated multi-item measures, and shared response influences remain possible. Finally, dangerousness, threat, police behavior, and intervention effectiveness were not assessed.

## 5 Conclusion

Across two model specifications, muscularity was consistently associated with perceived strength and resistance to blows for the male target and with strength, explosiveness, and resistance to blows for the female target. Additional findings, including male-target enthusiasm, depended on whether the interaction was included. No morality item survived correction. Training-group differences remained uncertain because experience was self-reported and highly unbalanced. The findings support further study of physical-capability impressions and require testing in realistic settings before educational applications are proposed.

## Data Availability

The raw data supporting the conclusions of this article will be made available by the authors, without undue reservation.
