# Supplementary material for: Muscularity and combatives training experience in social judgments among police cadets
Source: Front Psychol. 2026 Oct 21;17:1831520. doi: 10.3389/fpsyg.2026.1831520 (PMC13731258; doi:10.3389/fpsyg.2026.1831520)
Supplement: Supplementary file 1 [file Supplementary_file_1.DOCX]

**Table S1.** Adjusted combatives-experience contrasts for all outcomes

| **Target** | **Outcome** | **Experienced − inexperienced, B [95% CI]** | **Partial η²p** | **pHolm** |
| --- | --- | --- | --- | --- |
| Male | Law-abiding | −0.26 [−1.14, 0.62] | .001 | 1.000 |
| Male | Rule-compliance | −0.43 [−1.35, 0.49] | .003 | 1.000 |
| Male | Honesty | −0.43 [−1.25, 0.39] | .004 | 1.000 |
| Male | Justice | −0.48 [−1.33, 0.38] | .004 | 1.000 |
| Male | Friendliness | −0.56 [−1.50, 0.38] | .005 | 1.000 |
| Male | Enthusiasm | −0.21 [−1.19, 0.76] | .001 | 1.000 |
| Male | Approachability | −0.50 [−1.47, 0.46] | .004 | 1.000 |
| Male | Cooperativeness | −0.54 [−1.50, 0.42] | .004 | 1.000 |
| Male | Strength | 0.09 [−0.47, 0.65] | .000 | 1.000 |
| Male | Explosiveness | 0.23 [−0.44, 0.89] | .002 | 1.000 |
| Male | Fighting skill | 0.24 [−0.46, 0.94] | .002 | 1.000 |
| Male | Endurance | 0.29 [−0.53, 1.10] | .002 | 1.000 |
| Male | Coordination | 0.58 [−0.23, 1.38] | .007 | 1.000 |
| Male | Resistance to blows | 0.03 [−0.58, 0.64] | .000 | 1.000 |
| Female | Law-abiding | 0.12 [−0.76, 0.99] | .000 | 1.000 |
| Female | Rule-compliance | −0.22 [−1.13, 0.70] | .001 | 1.000 |
| Female | Honesty | −0.17 [−1.06, 0.73] | .000 | 1.000 |
| Female | Justice | −0.23 [−1.10, 0.65] | .001 | 1.000 |
| Female | Friendliness | 0.00 [−0.95, 0.95] | .000 | 1.000 |
| Female | Enthusiasm | −0.14 [−1.12, 0.84] | .000 | 1.000 |
| Female | Approachability | −0.07 [−1.13, 0.99] | .000 | 1.000 |
| Female | Cooperativeness | −0.06 [−1.00, 0.88] | .000 | 1.000 |
| Female | Strength | −0.30 [−1.11, 0.50] | .002 | 1.000 |
| Female | Explosiveness | −0.53 [−1.40, 0.34] | .005 | 1.000 |
| Female | Fighting skill | −0.64 [−1.58, 0.30] | .006 | 1.000 |
| Female | Endurance | −0.07 [−0.98, 0.84] | .000 | 1.000 |
| Female | Coordination | −0.22 [−1.09, 0.65] | .001 | 1.000 |
| Female | Resistance to blows | −0.27 [−1.22, 0.69] | .001 | 1.000 |

Note: B is the adjusted experienced-minus-inexperienced difference in 11-point scale units from the full models, averaged equally over the four image levels. Intervals are Wald 95% confidence intervals. pHolm adjusts 14 experience tests within each target set. All intervals include zero. Partial η²p = χ²/(χ² + 286).

**Table S2.** Nominal associations between participants’ self-rated strength and outcomes

| **Target** | **Outcome** | **B** | **95% Wald CI** | **Wald χ²** | ***p*** | **pHolm** |
| --- | --- | --- | --- | --- | --- | --- |
| Male | Law-abidingness | 0.214 | 0.069–0.359 | 8.394 | .004 | .049 |
| Male | Rule-compliance | 0.213 | 0.062–0.364 | 7.609 | .006 | .070 |
| Male | Honesty | 0.162 | 0.027–0.296 | 5.540 | .019 | .167 |
| Male | Justice | 0.197 | 0.056–0.337 | 7.553 | .006 | .070 |
| Male | Friendliness | 0.236 | 0.082–0.390 | 9.042 | .003 | .037 |
| Male | Approachability | 0.182 | 0.024–0.340 | 5.090 | .024 | .193 |
| Male | Cooperativeness | 0.195 | 0.038–0.353 | 5.907 | .015 | .151 |
| Female | Law-abidingness | 0.133 | 0.015–0.252 | 4.840 | .028 | .389 |
| Female | Honesty | 0.131 | 0.010–0.252 | 4.505 | .034 | .439 |

Note: B, unstandardized regression coefficient. pHolm adjusts the 14 self-rated-strength tests within each target set. Only male-target law-abidingness and friendliness remained below .05 after this adjustment.

**Table S3. Muscularity tests from models without the interaction**

| **Outcome** | **Male χ²(3)** | **Male raw p** | **Male pHolm** | **Female χ²(3)** | **Female raw p** | **Female pHolm** |
| --- | --- | --- | --- | --- | --- | --- |
| Law-abiding | 1.686 | 0.640 | 1.000 | 3.184 | 0.364 | 1.000 |
| Rule-compliance | 1.815 | 0.612 | 1.000 | 2.048 | 0.563 | 1.000 |
| Honesty | 8.185 | 0.042 | 0.296 | 5.480 | 0.140 | 1.000 |
| Justice | 6.540 | 0.088 | 0.529 | 9.538 | 0.023 | 0.206 |
| Friendliness | 9.648 | 0.022 | 0.174 | 4.782 | 0.188 | 1.000 |
| Enthusiasm | 15.798 | 0.001 | 0.014 | 2.399 | 0.494 | 1.000 |
| Approachability | 12.089 | 0.007 | 0.071 | 3.636 | 0.304 | 1.000 |
| Cooperativeness | 4.577 | 0.205 | 0.822 | 4.763 | 0.190 | 1.000 |
| Strength | 121.627 | <.001 | <.001 | 68.335 | <.001 | <.001 |
| Explosiveness | 25.031 | <.001 | <.001 | 33.361 | <.001 | <.001 |
| Fighting skill | 5.848 | 0.119 | 0.596 | 13.791 | 0.003 | 0.035 |
| Endurance | 2.810 | 0.422 | 1.000 | 10.402 | 0.015 | 0.154 |
| Coordination | 11.301 | 0.010 | 0.092 | 3.494 | 0.321 | 1.000 |
| Resistance to blows | 44.563 | <.001 | <.001 | 24.459 | <.001 | <.001 |

Note: Models retained muscularity, experience, and all seven covariates, with 289 residual degrees of freedom. Wald tests used maximum-likelihood dispersion. pHolm adjusts 14 muscularity tests within each target set. Comparison with Table 4 assesses sensitivity to model specification; these tests do not compare target sexes.
